# Evaluation of Angiogenic Factors (PlGF and sFlt-1) in Pre-eclampsia Diagnosis

**DOI:** 10.1055/s-0040-1713916

**Published:** 2020-11-30

**Authors:** Catherine Primo Nogueira de Sá, Mirela Foresti Jiménez, Marcos Wengrover Rosa, Ellen Machado Arlindo, Antonio Celso Koehler Ayub, Rodrigo Bernardes Cardoso, Régis Kreitchmann, Patrícia El Beitune

**Affiliations:** 1 Department of Gynecology and Obstetrics, Universidade Federal de Ciências da Saúde, Porto Alegre, RS, Brazil; 2 Obstetrics Service, Santa Casa de Misericórdia, Porto Alegre, RS, Brazil; 3 Obstetrics Service, Hospital Moinhos de Vento, Porto Alegre, RS, Brazil

**Keywords:** angiogenic factors, pre-eclampsia, PlGF, protein creatinine ratio, sFlt-1, fatores angiogênicos, pré-eclâmpsia, PlGF, relação proteína creatinina, sFlt-1

## Abstract

**Objective** Recent observations support the hypothesis that an imbalance between angiogenic factors has a fundamental role in the pathogenesis of pre-eclampsia and is responsible for the clinical manifestations of the disease. The goal of the present study was to evaluate the sensitivity, specificity, and the best accuracy level of Soluble fms-like tyrosine kinase-1 (sFlt-1), placental growth factor (PlGF), and sFlt-1/PlGF ratio in maternal serum and protein/creatinine ratio in urine sample to define the best cutoff point of these tests to discriminate between the patients with gestational hypertension and the patients with pre-eclampsia, to evaluate the possibility of using them as diagnostic methods.

**Methods** A prospective longitudinal study was performed, and blood samples were collected from 95 pregnant patients with hypertension to measure serum concentrations of biomarkers sFlt-1 and PlGF. Urine samples were collected for protein screening. Significance was set as *p* < 0.05.

**Results** The sFlt-1/PlGF ratio demonstrated a sensitivity of 57.5% and a specificity of 60% using 50.4 as a cutoff point. The test that showed the best accuracy in the diagnosis of pre-eclampsia was protein/creatinine ratio, with a sensitivity of 78.9% and a specificity of 70% using 0.4 as a cutoff point and showing an area under the receiver operating characteristic curve of 0.80 ( *p* < 0.001).

**Conclusion** No studied laboratory test proved to be fairly accurate for the diagnosis of pre-eclampsia, except for the protein/creatinine ratio. The evidence is insufficient to recommend biomarkers sFlt-1 and PlGF to be used for the diagnosis of pre-eclampsia.

## Resumo

**Objetivo** Pesquisas recentes sustentam a hipótese de que um desequilíbrio entre fatores angiogênicos desempenhe um papel fundamental na patogênese da pré-eclâmpsia e seja responsável pelas manifestações clínicas da doença. O objetivo do presente estudo foi avaliar a sensibilidade, a especificidade e o nível de melhor acurácia do Fator semelhante a tirosina quinase 1 (sFlt-1), Fator de crescimento placentário (PlGF), e relação sFlt-1/PlGF no soro materno e relação proteína/creatinina em amostra de urina e definir o melhor ponto de corte desses testes para distinguir pacientes com hipertensão gestacional daquelas com pré-eclâmpsia, a fim de avaliar a possibilidade de utilizá-los como métodos diagnósticos.

**Métodos** Foi realizado um estudo prospectivo longitudinal e foram coletadas amostras de sangue de 95 gestantes com hipertensão arterial para dosar as concentrações séricas dos biomarcadores sFlt-1 e PlGF. Amostras de urina foram coletadas para pesquisa de proteinúria. Foram consideradas significativas as diferenças com *p* < 0,05.

**Resultados** A relação sFlt-1/PlGF demonstrou sensibilidade de 57,5% e especificidade de 60% utilizando 50,4 como ponto de corte. O teste que apresentou a melhor acurácia no diagnóstico de pré-eclâmpsia foi a relação proteína/creatinina, com sensibilidade de 78,9% e especificidade de 70%, utilizando 0,4 como ponto de corte e demostrando uma área sob a curva receiver operating characteristic (ROC, na sigla em inglês) de 0,80 ( *p* < 0,001).

**Conclusão** Nenhum método de rastreamento isolado se mostrou com boa acurácia para o diagnóstico de pré-eclâmpsia, exceto a relação proteína/creatinina. As evidências são insuficientes para recomendar os biomarcadores sFlt-1 e PlGF como diagnóstico de pré-eclâmpsia.

## Introduction

The hypertensive disorders of pregnancy are a leading cause of maternal and perinatal mortality and morbidity worldwide, especially in developing countries, affecting 10% of pregnancies, and have been responsible for high costs to the health system. 1 2 3 Pre-eclampsia and gestational hypertension are characterized by the new onset of hypertension (> 140 mm Hg systolic or > 90 mm Hg diastolic) after 20 weeks of gestation. The next step is to define whether this represents pure gestational hypertension or pre-eclampsia. Pre-eclampsia is diagnosed by hypertension and the coexistence of one or more of the following conditions: proteinuria (urine protein/creatinine > 0.3 mg/mg or > 300 mg/day); maternal organ dysfunction (renal insufficiency, liver involvement, neurological complications, hematological complications); and uteroplacental dysfunction (fetal growth restriction). 4 5 Although often accompanied by new onset proteinuria, hypertension and other signs or symptoms of pre-eclampsia may present in some women in the absence of proteinuria. 6

The pathogenesis of pre-eclampsia involves deficient trophoblast invasion that is responsible for altered uterine blood flow and placental oxidative stress. 7 Recent observations support the hypothesis that altered expression of placental antiangiogenic factors is responsible for the clinical manifestations of the disease. The damaged placenta produces higher concentrations of Soluble fms-like tyrosine kinase-1 (sFlt-1), a soluble receptor for vascular endothelial growth factor (VEGF) and placental growth factor (PlGF) that is released into the maternal circulation and is involved in endothelial dysfunction. 8 9 10 11 Soluble fms-like tyrosine kinase-1 is an endogenous antiangiogenic protein that is made by the placenta and acts by binding and neutralizing the proangiogenic proteins VEGF and PlGF. Decreased concentrations of the circulating proangiogenic factor PlGF and increased concentrations of the antiangiogenic factor sFlt-1 have been observed in pre-eclamptic patients, suggesting that an imbalance between these factors has a fundamental role in the pathogenesis of pre-eclampsia. 12 13 14 Thereby, both sFlt-1 and PlGF have been suggested to be useful for the diagnosis of pre-eclampsia.

The goal of the present study was to evaluate the sensitivity, specificity, and the best accuracy level of sFlt-1, PlGF, sFlt-1/PlGF ratio in maternal serum and protein/creatinine ratio in urine sample to define the best cutoff point of these tests to discriminate between the patients with gestational hypertension and the patients with pre-eclampsia, to evaluate the possibility of using them as diagnostic methods. In addition, we evaluated the degree of association of 24-hour proteinuria with sFlt-1, PlGF, sFlt-1/PlGF ratio and protein/creatinine ratio.

## Methods

A prospective longitudinal study evaluated 95 pregnant women with hypertension in attendance at prenatal clinics and at the obstetric emergency of a tertiary university hospital in the south of Brazil (Maternidade Mário Totta – Santa Casa de Misericórdia de Porto Alegre, state of Rio Grande do Sul, Brazil) over a period of 12 months (October 2010 to October 2011). All included patients signed an informed consent form. The present study was approved by the Institutional Review Board (CEP UFCSPA 10–628).

The present study included pregnant women after 20 weeks of gestation with systolic blood pressure ≥ 140 mm Hg or diastolic blood pressure ≥ 90 mm Hg, measured according to a standard protocol, 15 16 and with ≥ 1 occurrences of protein on a dipstick or a protein/creatinine ratio ≥ 40 mg/mmol. These tests were considered screening methods. Thus, patients with positive screening had 24-hour proteinuria collection to confirm or exclude pre-eclampsia, considering this test as the evaluation parameter used in our institution for the diagnosis of the disease. Thereby, the 24-hour proteinuria was used to stratify patients into two groups, gestational hypertension and pre-eclampsia. The 24-hour collection was performed during hospitalization of the patient, under supervision of the nursing staff, following standard procedures established by institutional guidelines, which contributes to more accurate results. We excluded patients with diabetes mellitus and vascular disease prior to the pregnancy or preexisting kidney disease.

Blood samples were collected and serum concentrations of sFlt-1 and PlGF were measured at the time of the diagnosis. The Elecsys (Roche Diagnostics Brazil São Paulo, SP, Brazil) immunoassays for determination of sFlt-1 and PlGF and analysis of blood samples were performed at the central laboratory of the Santa Casa. Clinical information was verified through data collection during hospitalization, searching for maternal and gestational data, risk factors, gestational prognosis (eclampsia and HELLP Syndrome), and other relevant evaluation parameters (hyperuricemia, severe hypertension, proteinuria ≥ 5 g, fetal growth restriction). We made a separate analysis with the primigravida group. In addition, patients were stratified into two groups, early-onset pre-eclampsia (< 34 weeks of gestation) and late-onset pre-eclampsia (≥ 34 weeks of gestation).

The quantitative variables were described by mean and standard deviation (SD), or median and interquartile range (IQR). To compare averages between groups, the *t* -test was applied. For asymmetric variables, we used the Mann Whitney test. To compare proportions, the chi-squared test or the Fisher exact test was applied. Serum levels of sFlt-1 and PlGF, as well as biochemical parameters, were evaluated for sensitivity, specificity, and the optimal cutoff point by receiver operating characteristic (ROC) curve. The Spearman correlation coefficient (r) was used to evaluate the degree of association between the tests. Sample size was calculated considering an α of 0.05 and a β of 0.20 and setting the null hypothesis in an area under the ROC curve of 0.75. A total of 75 patients were necessary. Statistical analysis was performed using PASW Statistics for Windows, Version 18 (SPSS Inc., Chicago, IL, USA) and the significance level adopted was 0.05.

## Results

Table 1 shows maternal and gestational data, risk factors (chronic/preexisting hypertension, pre-eclampsia in a previous pregnancy and family history of pre-eclampsia) and some relevant evaluation parameters (uric acid > 6 mg/dL, systolic blood pressure ≥ 160 mm Hg or diastolic blood pressure ≥ 110 mm Hg, proteinuria > 5 g in 24 hours and fetal growth restriction). Besides, we can observe the screening methods, biomarkers sFlt-1 and PlGF, and 24-hour proteinuria levels. A total of 95 pregnant patients had blood samples collected to measure serum concentrations of biomarkers sFlt-1 and PlGF. One patient has been excluded from the analysis because there was no screening test performed, and another patient has been excluded because there was no registered value of PlGF. Thus, the statistical analysis included 93 pregnant patients with hypertension.

**Table 1:** Sample characterization of patients included in the study

Variables *	Total Sample ( *n* = 93)	Pre-eclampsia ( *n* = 73)	Gestational Hypertension ( *n* = 20)	*p-value*
Demographic data				
Maternal age (years old)	29.1 ± 7.7	29.5 ± 7.7	27.9 ± 7.8	0.422
Body mass index (kg/m ^2^ )	34.0 ± 6.7	34.1 ± 7.1	33.3 ± 4.4	0.666
Gestational data				
Gestational age at diagnosis (weeks)	34.2 ± 4.0	34.2 ± 4.4	34.1 ± 2.5	0.915
Nulliparity	39 (41.9)	31 (42.5)	8 (40.0)	1.000
Risk factors				
Chronic/preexisting hypertension	42 (45.2)	34 (46.6)	8 (40.0)	0.787
Preeclampsia in a previous pregnancy	6 (6.5)	5 (6.8)	1 (5.0)	1.000
Family history of preeclampsia	3 (3.2)	3 (4.1)	0 (0.0)	1.000
Evaluation parameters				
Uric acid ≥ 6 mg/dL	22 (23.9)	18 (24.7)	4 (21.1)	1.000
Severe blood pressure elevation	47 (50.5)	38 (52.1)	9 (45.0)	0.759
Proteinuria ≥ 5 g in 24 hours	1 (1.5)	1 (2.0)	0 (0.0)	1.000
Fetal growth restriction	12 (12.9)	8 (11.0)	4 (20.0)	0.280
Laboratory tests				
Protein/creatinine (mg/mmol)	0.47 (0.36–0.96)	0.54 (0.41–1.13)	0.33 (0.24–0.42)	< 0.001
Protein/creatinine ratio ≥ 0.4	62 (68.9)	56 (80.0)	6 (30.0)	< 0.001
Positive preeclampsia screening	70 (75.3)	60 (82.2)	10 (50.0)	0.007
24-hour proteinuria (g)	0.37 (0.29–0.57)	0.46 (0.34–0.64)	0.21 (0.15–0.27)	< 0.001
PlGF (pg/mL)	100 (62.3–235)	97.7 (62.3–235)	119 (56.7–283)	0.495
sFlt-1 (pg/mL)	5253 (2649–9071)	5469 (2703–9375)	3920 (1613–8574)	0.169
sFlt-1/PlGF ratio	55.3 (14.7–113)	58.7 (16.5–122)	34.3 (6.5–95.5)	0.258
Uric acid (mg/dL)	5.16 ± 1.30	5.21 ± 1.30	4.97 ± 1.31	0.475

Abbreviations: P1GF, placental growth factor; sFlt-1, Soluble fms-like tyrosine kinase-1.

* described by mean ± standard deviation, median (percentiles 25 - 75) or n (%).

Pre-eclampsia was confirmed in 73 patients (78.5%). A total of 29 (39.7%) patients had early-onset pre-eclampsia and 44 (60.3%) had late-onset disease. When evaluating the pre-eclampsia group and the gestational hypertension group, there was no association with nulliparity and no significant difference between early or late-onset pre-eclampsia. Only one patient developed eclampsia and HELLP syndrome (1.1%). Most of the pregnant patients were in the 3 ^rd^ trimester when they entered in the study, justifying higher values for the body mass index (BMI), which was calculated at the moment the patient was included and not in the beginning of pregnancy.

We analyzed laboratory tests individually and could observe a significant association of protein/creatinine ratio and 24-hour proteinuria with the diagnosis of pre-eclampsia. The biomarkers sFlt-1 and PlGF did not have a good accuracy for disease diagnosis. Demographic and gestational data, risk factors and other evaluation parameters did not have a significant association with pre-eclampsia in our study.

**Fig. 1:**
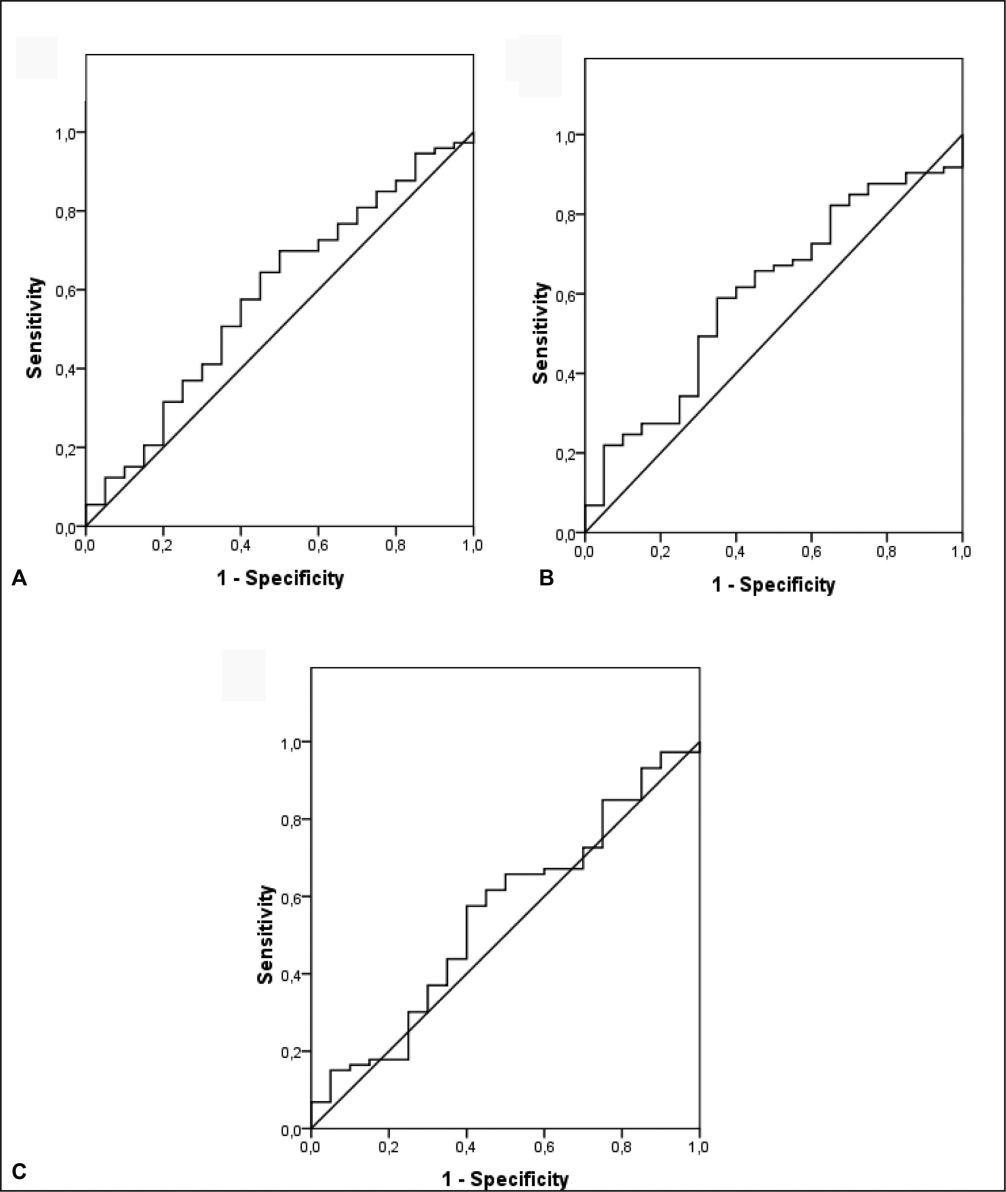
ROC curve of sFlt-1/PlGF ratio (A), sFlt-1 (B) and PlGF (C) in the diagnosis of preeclampsia.

**Fig. 2:**
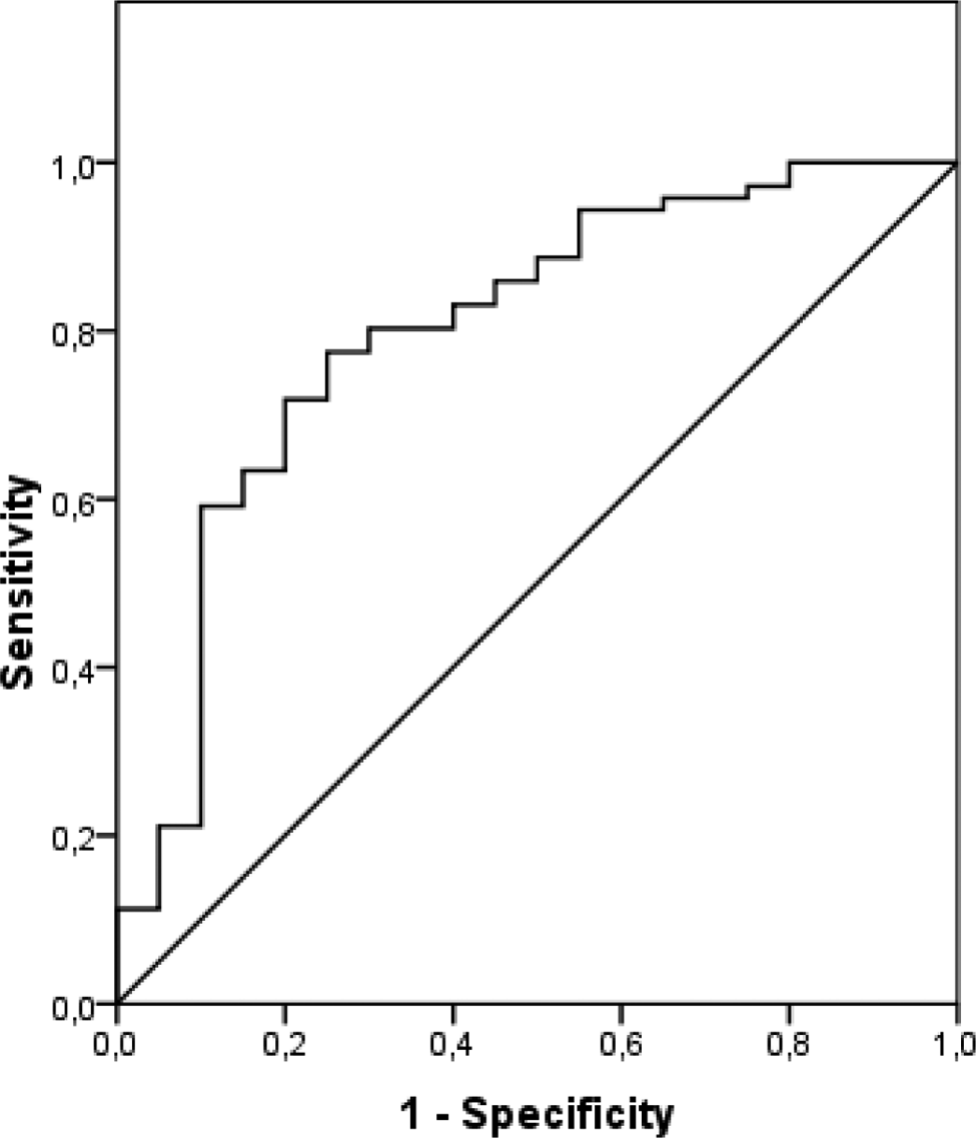
ROC curve of protein/creatinine ratio in preeclampsia diagnosis.

We determined the sensitivity and the specificity for different thresholds of a parameter, defining the best cutoff point for each test. Calculating the area under the ROC curve, we evaluated the performance of the studied tests ( Table 2 ). The sFlt-1/PlGF ratio demonstrated a sensitivity of 57.5% and a specificity of 60% using 50.4 as a cutoff point. The protein/creatinine ratio showed the best sensitivity (78.9%) and specificity (70%) using 0.4 as a cutoff point and was the test with the best accuracy level in pre-eclampsia diagnosis, showing an area under the ROC curve of 0.80 ( *p* < 0.001). In Figs. 1 and 2 , the sensitivity, specificity and the area under the ROC curve for the sFlt-1, PlGF, sFlt-1/PlGF ratio and protein/creatinine ratio can be observed.

**Table 2:** Evaluation of the best cutoff point and the area under the ROC curve of the studied tests for the diagnosis of pre-eclampsia, with their sensitivity and specificity

Tests	Sensitivity	Specificity	Cutoff point	AUC ROC	95%CI	*p-value*
sFlt-1/PlGF	57.5	60.0	> 50.4	0.58	0.44–0.73	0.258
sFlt-1	61.6	60.0	> 4671	0.60	0.46–0.74	0.169
PlGF	57.5	60.0	< 104.1	0.55	0.41–0.69	0.495
Protein/creatinine	78.9	70.0	> 0.40	0.80	0.68–0.92	< 0.001

Abbreviations: AUC, area under the curve; CI, confidence interval; P1GF, placental growth factor; ROC, receiver operating characteristic; sFlt-1, Soluble fms-like tyrosine kinase-1.

We used Spearman correlation to evaluate the degree of association between 24-hour proteinuria with sFlt-1, PlGF, sFlt-1/PlGF ratio and protein/creatinine ratio. For sFlt-1 (rs= 0.175; *p* = 0.163), PlGF (rs= - 0.066; *p* = 0.599) and sFlt-1/PlGF (rs= 0.107; *p* = 0.396), there was no degree of association. The only test that showed significant association with 24-hour proteinuria was protein/creatinine ratio, with a regular association (rs= 0.403; *p* = 0.001).

## Discussion

Several studies have evaluated the role of biochemical markers or a combination of biochemical and biophysical markers in the prediction of pre-eclampsia in the 1 ^st^ and 2 ^nd^ trimesters of pregnancy. Clinical, ultrasonographic, and laboratory parameters have been explored during early pregnancy as tools for predicting who will later develop pre-eclampsia. None of these, individually, have sufficient sensitivity and predictive values to be useful clinically, even among women at increased risk. 17 18 19 20 21 Regardless of the parameters used, screening for pre-eclampsia in low-risk women is associated with very low positive predictive values ranging from 8 to 33%. 22

The most studied antiangiogenic and proangiogenic markers have been sFlt-1 (soluble receptor for VEGF and PlGF) and PlGF. Studies show that lower concentrations of PlGF and higher concentrations of sFlt-1 during pregnancy confer an increased risk for the subsequent development of pre-eclampsia. Some have noted that maternal serum concentrations of these factors significantly separated healthy pregnant women and women with pre-eclampsia, showing the value of these markers in the prediction of pre-eclampsia and in the differential diagnosis of patients with atypical presentations of the disease. In addition, in high-risk women, the sFlt-1/PlGF ratio is altered prior to pre-eclampsia onset. 23 24 25 26

Recent studies have evaluated the performance of a newly developed assay for biomarkers PlGF and sFlt-1, which has been studied for the prediction, diagnosis and prognosis of patients with pre-eclampsia. A study conducted by Hagmann et al 27 showed that in early onset pre-eclampsia, the sFlt-1/PlGF ratio changes 11 weeks before delivery. Zeisler et al 28 observed that a sFlt-1/PlGF ratio < 38 rules out pre-eclampsia, irrespective of gestational age, for at least 1 week. In women with an elevated sFlt-1/PlGF ratio > 85 (early-onset pre-eclampsia) or > 110 (late-onset pre-eclampsia), the diagnosis of pre-eclampsia or placenta related disorders is highly likely. Severely elevated sFlt-1/PlGF ratios (> 655 at < 34 weeks; > 201 at ≥ 34 weeks) are associated closely with the need to deliver within 48 hours. 29

Many studies evaluated biomarkers for the prediction of pre-eclampsia. Different from those studies, we evaluated angiogenic factors for the purpose of diagnostic correlation with pre-eclampsia. In a longitudinal prospective study, with adherence to methodological criteria, reviewed in detail, excluding any pregnant woman who could be configured to bias the results analysis, we studied the role of biomarkers PlGF and sFlt-1 in the diagnostic of pre-eclampsia. The present study demonstrated the cutoff point for PlGF (104.1) and for sFlt-1 (4671). Considering these biomarkers, the sFlt-1/PlGF ratio revealed the best association with pre-eclampsia diagnosis using 50.4 as a cutoff point. However, our results showed that sFlt-1, PlGF, and sFlt-1/PlGF ratio did not have good diagnostic accuracy.

Although the literature presents favorable evidence, there are many controversies on the benefits that these biomarkers may provide in the assessment of pre-eclampsia. A study conducted at Cambridge University showed that higher levels of sFlt-1 were not associated with the risk of pre-eclampsia but were associated with a reduced risk of delivery of a small for gestational age infant, spontaneous preterm birth, and stillbirth associated with abruption or growth restriction. 30 A case-control study showed that sFlt-1 levels had low capacity to discriminate between healthy patients and pre-eclampsia patients. 31 A systematic review accomplished at Oxford University demonstrated that a 3 ^rd^ trimester increase in sFlt-1 and decrease in PlGF levels were associated with pre-eclampsia, specifically severe disease; however, the authors concluded that the evidence is insufficient to recommend these biomarkers to be used for screening. 32

The most reasonable conclusions seem to be that determination of sFlt-1/PlGF ratio can serve as an aid in the diagnosis of hypertensive disorders in pregnancy. The performance of maternal levels of these factors, especially on early onset pre-eclampsia, could be further improved by combining several markers. Combining biomarkers with maternal history, mean blood pressure and uterine artery Doppler achieves a detection rate of ∼ 90% of cases to develop pre-eclampsia. 33

Some important considerations of our study are presented below. When our study was initiated, in October 2010, the protein/creatinine ratio was considered a screening method. Thus, pregnant women with positive screening (≥ 1 on dipstick or protein/creatinine ratio ≥ 40 mg/mmol) were considered inclusion criteria of the study, requiring the 24-hour proteinuria collection to define or exclude the diagnosis of pre-eclampsia. We used a cutoff level of 0.4 mg/mg for protein/creatinine ratio according to previous studies performed at our institution, because this cutoff point showed the best accuracy level for pre-eclampsia screening in our pregnant women population. Midrange protein/creatinine ratio (0.3 mg/mg) had poor sensitivity and specificity in our study. In another hospital, located in the same city of our study, they found that hypertensive pregnant women with a protein/creatinine ratio ≥ 0.3 mg/mg had worse maternal and perinatal outcomes than those with a protein/creatinine ratio < 0.3 mg/mg. 34 The latest guidelines already consider the use of protein/creatinine ratio as part of criteria for the diagnosis of pre-eclampsia, but many authors and institutions continue to use and consider a full 24-hour urine test for accurate results. 35 The existing evidence is not, however, sufficient to determine how the protein/creatinine ratio should be used in clinical practice, owing to the heterogeneity in test accuracy and prevalence across studies. 36

We made a separate analysis with the primigravida group to assess the correlation with the diagnosis of pre-eclampsia; however, in our study, there was no association with nulliparity. Besides, patients were stratified into 2 groups, early-onset pre-eclampsia (< 34 weeks of gestation) and late-onset pre-eclampsia (≥ 34 weeks of gestation) and, similarly, there was no significant difference between the groups. Maybe these associations could be observed if we had a larger sample.

Although some criteria are currently no longer considered as severity, when analyzed together, they may have clinical significance. Therefore, we evaluated hyperuricemia, severe hypertension, proteinuria ≥ 5 g and fetal growth restriction as relevant parameters. The evaluation of these parameters did not show a significant difference when comparing pre-eclampsia and gestational hypertension. Twenty-four-hour proteinuria > 5 g has not been considered as a maternal prognosis to indicate pregnancy resolution. 6

The BMI was calculated at the entry of the study, not in the beginning of the pregnancy, justifying higher values for this ratio. Furthermore, as the sample comprises hypertensive pregnant women, we could observe higher values for the BMI, since obesity is a risk factor for hypertension. Moreover, in recent decades, there has been an increase in weight in the general population. The growing prevalence of obesity is increasingly recognized as one of the most important risk factors for the development of hypertension. 37

Concluding, we can observe that there is considerable heterogeneity among reports. There are differences in the analyte and storage conditions, in the gestational periods selected for blood sampling, and in inclusion and exclusion criteria. Some reports include women with risk factors for pre-eclampsia, whereas others excluded this group; some of the study population was exclusively nulliparous women, whereas all parities were included in others. The evidence is neither strong enough nor sufficient to recommend PlGF and sFlt-1 to be used for pre-eclampsia diagnosis or to screen women at risk to develop the disease. Therefore, the use of proangiogenic and antiangiogenic factors in the assessment of pre-eclampsia is a subject of controversy and is currently under investigation. Prospective studies employing rigorous laboratory and study design criteria are needed to determine the clinical usefulness of these tests.

## Conclusion

In summary, in our research, no studied laboratory test proved to be fairly accurate for the diagnosis of pre-eclampsia, except for the protein/creatinine ratio. The evidence is insufficient to recommend sFlt-1 and PlGF to be used for the diagnosis of pre-eclampsia. The identification of biomarkers that can contribute to early detection of pre-eclampsia is essential to apply better surveillance and treatment protocols. Besides, demonstrating the clinical utility of these angiogenic markers could affect the management decisions of the obstetrician, improve health outcomes, and reduce costs to the healthcare system.

## References

[1] World Health Organization The World Health Report 2005: make every mother and child count [Internet] Geneva WHO 2005[cited 2019 Nov 17]. Available from:https://www.who.int/whr/2005/whr2005_en.pdf?ua=1

[2] American College of Obstetricians and Gynecologists Task Force on Hypertension in Pregnancy Hypertension in pregnancy. Report of the American College of Obstetricians and Gynecologists' Task Force on Hypertension in Pregnancy Obstet Gynecol 2013 122 05 1122 1131 10.1097/01.AOG.0000437382.03963.88 24150027

[3] Duley L The global impact of pre-eclampsia and eclampsia Semin Perinatol 2009 33 03 130 137 10.1053/j.semperi.2009.02.010 19464502

[4] Canadian Hypertensive Disorders of Pregnancy (HDP) Working Group Canadian Hypertensive Disorders of Pregnancy HDP Working Group Magee L A Pels A Helewa M Rey E von Dadelszen P The hypertensive disorders of pregnancy (29.3) Best Pract Res Clin Obstet Gynaecol 2015 29 05 643 657 10.1016/j.bpobgyn.2015.04.001 26141795

[5] Tranquilli A L Dekker G Magee L Roberts J Sibai B M Steyn W The classification, diagnosis and management of the hypertensive disorders of pregnancy: A revised statement from the ISSHP Pregnancy Hypertens 2014 4 02 97 104 10.1016/j.preghy.2014.02.001 26104417

[6] American College of Obstetricians and Gynecologists ACOG Practice Bulletin No. 202: gestational hypertension and preeclampsia Obstet Gynecol 2019 133 01 e1 e25 10.1097/AOG.0000000000003018 30575675

[7] de Oliveira L G Karumanchi A Sass N [Preeclampsia: oxidative stress, inflammation and endothelial dysfunction] Rev Bras Ginecol Obstet 2010 32 12 609 616 10.1590/S0100-72032010001200008 21484030

[8] Kopcow H D Karumanchi S A Angiogenic factors and natural killer (NK) cells in the pathogenesis of preeclampsia J Reprod Immunol 2007 76(1-2):23 29 10.1016/j.jri.2007.03.018 17490755 PMC2147723

[9] Silasi M Cohen B Karumanchi S A Rana S Abnormal placentation, angiogenic factors, and the pathogenesis of preeclampsia Obstet Gynecol Clin North Am 2010 37 02 239 253 10.1016/j.ogc.2010.02.013 20685551

[10] Wang A Rana S Karumanchi S A Preeclampsia: the role of angiogenic factors in its pathogenesis Physiology (Bethesda) 2009 24 147 158 10.1152/physiol.00043.2008 19509125

[11] Lam C Lim K H Karumanchi S A Circulating angiogenic factors in the pathogenesis and prediction of preeclampsia Hypertension 2005 46 05 1077 1085 10.1161/01.HYP.0000187899.34379.b0 16230516

[12] Maynard S E Karumanchi S A Angiogenic factors and preeclampsia Semin Nephrol 2011 31 01 33 46 10.1016/j.semnephrol.2010.10.004 21266263 PMC3063446

[13] Maynard S Epstein F H Karumanchi S A Preeclampsia and angiogenic imbalance Annu Rev Med 2008 59 61 78 10.1146/annurev.med.59.110106.214058 17937587

[14] Kulkarni A V Mehendale S S Yadav H R Kilari A S Taralekar V S Joshi S R Circulating angiogenic factors and their association with birth outcomes in preeclampsia Hypertens Res 2010 33 06 561 567 10.1038/hr.2010.31 20224572

[15] Atherosclerosis, Hypertension, and Obesity in the Young Committee of the American Heart Association Council on Cardiovascular Disease in the Young Alpert B McCrindle B Daniels S Dennison B Hayman L Jacobson M Recommendations for blood pressure measurement in human and experimental animals; part 1: blood pressure measurement in humans Hypertension 2006 48 01 e3, author reply e5 10.1161/01.HYP.0000229661.06235.08 16769991

[16] Report of the National High Blood Pressure Education Program Working Group on High Blood Pressure in Pregnancy Am J Obstet Gynecol 2000 183 01 S1 S22 10.1067/mob.2000.107928 10920346

[17] Baweja S Kent A Masterson R Roberts S McMahon L P Prediction of pre-eclampsia in early pregnancy by estimating the spot urinary albumin: creatinine ratio using high-performance liquid chromatography BJOG 2011 118 09 1126 1132 10.1111/j.1471-0528.2011.02960.x 21481153

[18] EBM CONNECT Collaboration Kleinrouweler C E Wiegerinck M MJ Ris-Stalpers C Bossuyt P MM van der Post J AM von Dadelszen P Accuracy of circulating placental growth factor, vascular endothelial growth factor, soluble fms-like tyrosine kinase 1 and soluble endoglin in the prediction of pre-eclampsia: a systematic review and meta-analysis BJOG 2012 119 07 778 787 10.1111/j.1471-0528.2012.03311.x 22433027

[19] Di Lorenzo G Ceccarello M Cecotti V Ronfani L Monasta L Vecchi Brumatti L First trimester maternal serum PIGF, free β-hCG, PAPP-A, PP-13, uterine artery Doppler and maternal history for the prediction of preeclampsia Placenta 2012 33 06 495 501 10.1016/j.placenta.2012.03.003 22459245

[20] Eunice Kennedy Shriver National Institute of Child Health and Human Development (NICHD) Maternal-Fetal Medicine Units (MFMU) Network Myatt L Clifton R G Roberts J M Spong C Y Hauth J C Varner M W First-trimester prediction of preeclampsia in nulliparous women at low risk Obstet Gynecol 2012 119 06 1234 1242 10.1097/AOG.0b013e3182571669 22617589 PMC3360523

[21] Masoura S Kalogiannidis I A Gitas G Goutsiolis A Koiou E Athanasiadis A Vavatsi N Biomarkers in pre-eclampsia: a novel approach to early detection of the disease J Obstet Gynaecol 2012 32 07 609 616 10.3109/01443615.2012.709290 22943702

[22] Espinoza J Recent biomarkers for the identification of patients at risk for preeclampsia: the role of uteroplacental ischemia Expert Opin Med Diagn 2012 6 02 121 130 10.1517/17530059.2012.659726 23480655

[23] Moore Simas T A Crawford S L Solitro M J Frost S C Meyer B A Maynard S E Angiogenic factors for the prediction of preeclampsia in high-risk women Am J Obstet Gynecol 2007 197 03 2440 2.44E10 10.1016/j.ajog.2007.06.030 17826405

[24] Erez O Romero R Espinoza J Fu W Todem D Kusanovic J P The change in concentrations of angiogenic and anti-angiogenic factors in maternal plasma between the first and second trimesters in risk assessment for the subsequent development of preeclampsia and small-for-gestational age J Matern Fetal Neonatal Med 2008 21 05 279 287 10.1080/14767050802034545 18446652 PMC2846114

[25] Leaños-Miranda A Campos-Galicia I Isordia-Salas I Ayala-Méndez J A Ulloa-Aguirre A Changes in circulating concentrations of soluble fms-like tyrosine kinase-1 and placental growth factor measured by automated electrochemiluminescence immunoassays methods are predictors of preeclampsia J Hypertens 2012 30 11 2173 2181 10.1097/HJH.0b013e328357c0c9 22902831

[26] Verlohren S Galindo A Schlembach D Zeisler H Herraiz I Moertl M G An automated method for the determination of the sFlt-1/PIGF ratio in the assessment of preeclampsia Am J Obstet Gynecol 2010 202 02 1610 1.61E13 10.1016/j.ajog.2009.09.016 19850276

[27] Hagmann H Thadhani R Benzing T Karumanchi S A Stepan H The promise of angiogenic markers for the early diagnosis and prediction of preeclampsia Clin Chem 2012 58 05 837 845 10.1373/clinchem.2011.169094 22431894

[28] Zeisler H Llurba E Chantraine F Vatish M Staff A C Sennström M Predictive value of the sFlt-1:PlGF ratio in women with suspected preeclampsia N Engl J Med 2016 374 01 13 22 10.1056/NEJMoa1414838 26735990

[29] Stepan H Herraiz I Schlembach D Verlohren S Brennecke S Chantraine F Implementation of the sFlt-1/PlGF ratio for prediction and diagnosis of pre-eclampsia in singleton pregnancy: implications for clinical practice Ultrasound Obstet Gynecol 2015 45 03 241 246 10.1002/uog.14799 25736847 PMC4369131

[30] Smith G CS Crossley J A Aitken D A Jenkins N Lyall F Cameron A D Circulating angiogenic factors in early pregnancy and the risk of preeclampsia, intrauterine growth restriction, spontaneous preterm birth, and stillbirth Obstet Gynecol 2007 109 06 1316 1324 10.1097/01.AOG.0000265804.09161.0d 17540803

[31] Srinivas S K Larkin J Sammel M D Appleby D Bastek J Andrella C M The use of angiogenic factors in discriminating preeclampsia: are they ready for prime time? J Matern Fetal Neonatal Med 2010 23 11 1294 1300 10.3109/14767051003677988 20482287

[32] Widmer M Villar J Benigni A Conde-Agudelo A Karumanchi S A Lindheimer M Mapping the theories of preeclampsia and the role of angiogenic factors: a systematic review Obstet Gynecol 2007 109 01 168 180 10.1097/01.AOG.0000249609.04831.7c 17197602

[33] Scazzocchio E Figueras F Contemporary prediction of preeclampsia Curr Opin Obstet Gynecol 2011 23 02 65 71 10.1097/GCO.0b013e328344579c 21386680

[34] Martins-Costa S H Vettorazzi J Valério E Maurmann C Benevides G Hemessath M Protein creatinine ratio in random urine sample of hypertensive pregnant women: maternal and perinatal outcomes Hypertens Pregnancy 2011 30 03 331 337 10.3109/10641950903454564 21174587

[35] Papanna R Mann L K Kouides R W Glantz J C Protein/creatinine ratio in preeclampsia: a systematic review Obstet Gynecol 2008 112 01 135 144 10.1097/AOG.0b013e3181778cfc 18591319

[36] Morris R K Riley R D Doug M Deeks J J Kilby M D Diagnostic accuracy of spot urinary protein and albumin to creatinine ratios for detection of significant proteinuria or adverse pregnancy outcome in patients with suspected pre-eclampsia: systematic review and meta-analysis BMJ 2012 345 e4342 10.1136/bmj.e4342 22777026 PMC3392077

[37] Narkiewicz K Obesity and hypertension–the issue is more complex than we thought Nephrol Dial Transplant 2006 21 02 264 267 10.1093/ndt/gfi290 16311261

